# Machine learning-driven development of a stratified CES-D screening system: optimizing depression assessment through adaptive item selection

**DOI:** 10.1186/s12888-025-06693-8

**Published:** 2025-03-26

**Authors:** Ruo-Fei Xu, Zhen-Jing Liu, Shunan Ouyang, Qin Dong, Wen-Jing Yan, Dong-Wu Xu

**Affiliations:** 1 School of Mental Health, Wenzhou Medical University, Wenzhou, China; 2 Zhejiang Provincial Clinical Research Centre for Mental Health, Affiliated Kangning Hospital, Wenzhou Medical University, Wenzhou, 325000 China

**Keywords:** Depression screening, Stratified screening, Machine learning, CES-D, Recursive Feature Elimination, Predictive modeling

## Abstract

**Objective:**

To develop a stratified screening tool through machine learning approaches for the Center for Epidemiologic Studies Depression Scale (CES-D-20) while maintaining diagnostic accuracy, addressing the efficiency limitations in large-scale applications.

**Methods:**

Data were derived from the Chinese Psychological Health Guard Project (primary sample: *n* = 179,877; age 9–18) and China Labor-force Dynamics Survey (validation samples across age spans). We employed a two-stage machine learning approach: first applying Recursive Feature Elimination with multiple linear regression to identify core predictive items for total depression scores, followed by logistic regression for optimizing depression classification (CES-D ≥ 16). Model performance was systematically evaluated through discrimination (ROC analysis), calibration (Brier score), and clinical utility analyses (decision curve analysis), with additional validation using random forest and support vector machine algorithms across independent samples.

**Results:**

The resulting stratified screening system consists of an initial four-item rapid screening layer (encompassing emotional, cognitive, and interpersonal dimensions) for detecting probable depression (AUC = 0.982, sensitivity = 0.945, specificity = 0.926), followed by an enhanced assessment layer with five additional items. Together, these nine items enable accurate prediction of the full CES-D-20 total score (R^2^ = 0.957). This stratified approach demonstrated robust generalizability across age groups (R^2^ > 0.94, accuracy > 0.91) and time points. Calibration analyses and decision curve analyses confirmed optimal clinical utility, particularly in the critical risk threshold range (0.3–0.6).

**Conclusions:**

This study contributes to the refinement of CES-D by developing a machine learning-derived stratified screening version, offering an efficient and reliable approach that optimizes assessment burden while maintaining excellent psychometric properties. The stratified design makes it particularly valuable for large-scale mental health screening programs, enabling efficient risk stratification and targeted assessment allocation.

**Supplementary Information:**

The online version contains supplementary material available at 10.1186/s12888-025-06693-8.

## Introduction

Depression represents a significant global health challenge, affecting a substantial portion of the global population and placing substantial burden on healthcare systems. The Center for Epidemiologic Studies Depression Scale (CES-D-20) has been widely adopted as a reliable screening tool for depressive symptoms across diverse populations [1, 2]. However, in large-scale mental health screenings and epidemiological studies, the length of assessment tools can present practical challenges, potentially affecting response rates and data quality [3]. Recent advances in machine learning techniques offer promising opportunities to optimize traditional screening instruments while maintaining their diagnostic accuracy. This optimization is particularly crucial in contemporary healthcare settings, where efficient and accurate depression screening tools are essential for early intervention and treatment planning.

### Traditional methods for scale simplification

Previous efforts to simplify depression screening tools have primarily relied on classical test theory and factor analysis approaches. Early attempts to abbreviate the CES-D-20 resulted in several shortened versions, including the 10-item CES-D-10 [4] and the 8-item CES-D-8 [5], which demonstrated acceptable internal consistency (α = 0.73 to 0.84). Building on these efforts, Baron et al. [6] employed confirmatory factor analysis to create a 10-item version (CESD-10) that maintained good diagnostic accuracy (AUC = 0.81 to 0.94) while significantly reducing respondent burden. Similar approaches have been applied to other depression screening tools: Kroenke et al. [7] developed the PHQ-2 from the original PHQ-9 using classical psychometric methods, achieving a sensitivity of 83% and specificity of 92% for major depression. Traditional approaches have effectively shortened lengthy questionnaires through rigorous statistical analyses, successfully maintaining reliability while reducing respondent burden. These traditional methods tend to analyze items in isolation, often assuming similar importance across all items without fully capturing their varying contributions to depression assessment. Additionally, most shortened versions primarily serve screening purposes, leading clinicians to seek additional assessment tools when more detailed diagnostic information is needed.

### Machine learning for scale simplification

Machine learning techniques demonstrate promising potential in optimizing mental health screening instruments, and despite challenges in symptom variations and clinical judgment [8], have proven effective in psychiatric scale simplification.Several landmark studies illustrate this progression from basic dimensional reduction to sophisticated ensemble methods. In cross-cultural contexts, Wang et al. [9] analyzed GAD-7 scale data from 47,484 participants across six countries using the Riskslim algorithm, demonstrating that a carefully selected three-item version could achieve optimal balance between brevity and diagnostic accuracy while maintaining cross-cultural validity. For more complex instruments, Yu et al. [10] employed Support Vector Classification to reduce the Symptom Checklist-90 to 29 items while maintaining a high reliability coefficient of 0.95, while Shen et al. [11] combined Random Forest and Gradient Boosting Machine algorithms to successfully compress the Hypomania Checklist-32 to 8 items (AUC = 0.97) for adolescent populations. Most recently, advanced ensemble approaches have shown even greater potential, as demonstrated by Ding et al. [12], who integrated Shapley additive explanation with backpropagation neural networks to identify six key indicators from 25 features, achieving exceptional diagnostic accuracy (C-index = 0.9749) in external validation.

### Research gap

Current approaches to scale reduction present several key limitations that warrant attention. First, there remains a critical need for systematic quantification of the relationship between item quantity and explanatory power in depression screening tools. While existing methods effectively identify important items, they typically lack a precise understanding of how each additional item incrementally contributes to the scale's predictive accuracy. This understanding is crucial for making evidence-based decisions about optimal scale length rather than relying on predetermined cutoffs or arbitrary item selection criteria. Moreover, a fundamental limitation of existing shortened versions of depression screening tools is their one-size-fits-all approach, serving either rapid screening or comprehensive assessment purposes, but rarely both. A more flexible approach is needed to meet the varying demands of clinical practice and research, where both rapid screening tools and comprehensive assessments serve essential roles.

### The present study

To address the identified methodological limitations in depression scale reduction, this study proposes an innovative dual-component approach that combines advanced machine learning techniques with clinical practicality. Our research introduces a novel methodological framework that systematically bridges the gap between brief screening needs and comprehensive diagnostic assessment while maintaining measurement precision across the depression severity spectrum.

The first component of our approach centers on the application of Recursive Feature Elimination (RFE) within a machine learning framework to systematically examine and quantify the relationship between item quantity and explanatory power in depression assessment. Unlike traditional methods that rely on predetermined thresholds or arbitrary cutoff points, RFE enables a data-driven approach to understanding how each item contributes to the overall predictive accuracy of the scale. This method iteratively removes features while monitoring changes in model performance, providing empirical evidence for identifying optimal reduction points where additional items yield diminishing returns.

Building upon this methodological foundation, our second innovation introduces an integrated hierarchical screening framework that systematically synthesizes rapid screening capabilities with comprehensive assessment potential. This framework implements a sophisticated two-tiered approach that begins with a first-tier screening comprising a minimal set of maximally predictive core items for detecting depressive symptoms. These core items are strategically selected as a subset of the more comprehensive second-tier assessment, enabling seamless transition between screening levels while maintaining measurement continuity. The framework's design ensures that when first-tier screening results indicate low depression risk, the assessment process can conclude efficiently. However, when first-tier screenings suggest elevated risk, the framework seamlessly transitions to the completion of remaining second-tier items, with responses from both tiers integrated to generate a comprehensive depression severity score.

The integration of these two components addresses several critical limitations in current approaches. First, it provides an empirically-driven method for determining optimal scale length based on concrete evidence rather than arbitrary decisions. Second, it offers a flexible assessment system that adapts to varying clinical needs while maintaining measurement precision. Third, it enables efficient resource allocation by minimizing unnecessary comprehensive assessments while ensuring thorough evaluation when warranted. Most importantly, it maintains measurement continuity across different assessment phases, addressing a significant limitation of existing two-stage screening approaches.

## Method

### Participants and procedures

This study utilized two large-scale databases: the Chinese Psychological Health Guard Project (CPHG) as the primary database and the China Labor-force Dynamics Survey (CLDS) for cross-age validation.

The CPHG is a large-scale, multi-center cohort study evaluating depression and suicidal ideation among vulnerable children and adolescents aged 9–18 years. Initial data collection commenced in October 2022 (baseline, T1), followed by a six-month follow-up survey (T2). After data cleaning, the effective sample size of left-behind children at T1 was 179,877 (48.7% females; 61.9% rural residents), with 133,904 completing T2 assessment. An additional 48,128 children from single-parent families were assessed at T1. Data collection was conducted across 569 centers using a dedicated smartphone application for CES-D administration, demonstrating excellent internal consistency (Cronbach's α = 0.94). The study received ethical approval (NCPP 2022002), with electronic informed consent obtained from all guardians.

The CLDS database provided comprehensive age-span validation data through a longitudinal design. Participants were stratified into four age groups: adolescents (15–18 years; T1: *n* = 1,075; T2: *n* = 742), young adults (19–30 years; T1: *n* = 3,207; T2: *n* = 2,052), middle-aged adults (31–65 years; T1: *n* = 15,758; T2: *n* = 12,697), and older adults (≥ 66 years; T1: *n* = 948; T2: *n* = 955). This diverse age distribution enabled thorough evaluation of the model's predictive efficacy across different life stages.

Both the CPHG and CLDS are publicly available databases with detailed demographic, inclusion/exclusion criteria, and sampling procedures documented in previous studies. the clds used a multi-stage pps sampling method and tracking across multiple waves to capture social changes in china. detailed procedures and weight calculations are available through the clds data portal (cphg: https://doi.org/10.1038/s41597-024-03130-5, clds: https://doi.org/10.1177/2397200917735796).

### Data analysis

#### Data preprocessing

The study utilized T1 data of left-behind children and adolescents (*n* = 179,877) from the CPHG database as the primary dataset for model training and development. Initially, CES-D total scores were calculated for each participant, and observations with missing values were removed to ensure data quality. The dataset was randomly split into training and testing sets in a 7:3 ratio to ensure scientific rigor in model development and evaluation independence, effectively preventing overfitting. The remaining independent samples were used for external validation, with missing values similarly processed to ensure data integrity and reliability of analytical results.

#### Feature selection and modeling

Feature selection was conducted using Recursive Feature Elimination (RFE) with linear regression as the base estimator. To enhance robustness, ten-fold cross-validation was applied to evaluate model performance (R^2^) across different feature subsets (1–20). The optimal number of features was determined using a data-driven approach by identifying the inflection point where the marginal gain in R^2^ significantly diminished, ensuring a balance between model simplicity and predictive accuracy [13]. Traditional psychometric approaches, such as factor analysis (FA) and item response theory (IRT), quantify item contributions through factor loadings and discrimination parameters, offering essential insights into the internal structure and latent construct of depression. However, these methods primarily focus on measurement validity rather than direct predictive performance. In contrast, our approach, based on Recursive Feature Elimination (RFE), selects items purely for predictive purposes, optimizing their ability to estimate total CES-D scores. By iteratively refining item selection based on predictive accuracy, our method does not aim to model the latent structure of depression but rather to enhance screening efficiency in large-scale applications. A relationship plot between the number of selected features and R^2^ scores was generated to visualize this trade-off. Additionally, feature rankings were aggregated across RFE iterations to identify the most predictive variables for the CES-D total score. The final predictive model, based on the minimal feature subset at the inflection point, was validated using ten-fold cross-validation to assess its performance [14]. For classification model training, random undersampling was first applied to address the imbalance between depressive and non-depressive cases, ensuring equal representation of both categories in the training data. This step was crucial for developing a model equally sensitive to both positive and negative cases, preventing potential bias against minority groups that could arise from class imbalance. Following balanced sampling, ROC curve analysis was employed to assess the discriminative ability of cumulative feature combinations [15]. Using CES-D total score ≥ 16 as the diagnostic threshold for clinical depressive symptoms, results were converted into binary variables. Based on feature importance rankings, progressively expanding logistic regression models were constructed, with their discriminative ability quantified through AUC values [16]. For the brief screening layer, we selected the four highest-ranked items from the RFE results. The effectiveness of this four-item combination was then thoroughly evaluated using multiple performance metrics, including accuracy, F1 score, specificity, recall, and precision. The Youden index was used to optimize the balance between sensitivity and specificity, determining the optimal classification threshold.

#### Model comparison

To compare different classification models' performance, the study trained random forest and support vector machine algorithms in addition to logistic regression. To ensure robust and reliable model evaluation, five-fold cross-validation was repeated five times, generating 25 independent assessments of model performance. This repeated cross-validation approach minimizes the impact of random data partitioning and provides sufficient samples for statistical comparison of model performance. Using the Area Under the ROC Curve (AUC) as the primary evaluation metric, the study comprehensively examined multiple indicators including sensitivity, specificity, accuracy, precision, and F1 score to thoroughly assess model performance. One-way ANOVA was used to test for significant differences in AUC values between models, with Tukey's HSD test for multiple comparisons when *p* < 0.05 to clarify specific inter-model differences. The Youden index was used to determine optimal classification thresholds, providing clear decision criteria for clinical applications. The best-performing classification model was ultimately selected.

#### External validation

To verify the model's generalizability and predictive efficacy, this study employed a multi-sample external validation strategy. The validation samples included child and adolescent data from the CPHG project (left-behind children and children from single-parent families) and samples across different age groups from adolescence to elderly in the CLDS database, encompassing both baseline and follow-up measurement data. This multi-sample design ensured comprehensive validation, covering both special populations and general populations across different age groups.

For the external validation, we implemented a systematic evaluation approach encompassing both regression and classification models. The nine-feature linear regression model was primarily assessed through its variance explanation capability, with R^2^ values used to examine the model's explanatory power across different populations. The four-feature classification model underwent a comprehensive performance evaluation scheme that included three key components: discrimination metrics (accuracy, sensitivity, specificity, precision, F1 score, and AUC values), calibration plot analysis to evaluate the concordance between predicted probabilities and actual risks, and clinical decision curve analysis to assess net clinical benefit across different decision thresholds. This multi-faceted validation approach provided a thorough assessment of the models' performance and clinical utility across diverse populations.All validation samples utilized the CES-D scale as the assessment tool and employed uniform statistical analysis methods, ensuring the comparability of evaluation results. This multidimensional validation strategy not only verified the model's statistical performance but also provided empirical evidence for its clinical value in practical applications.

### Statistical tools

**Fig. 1:**
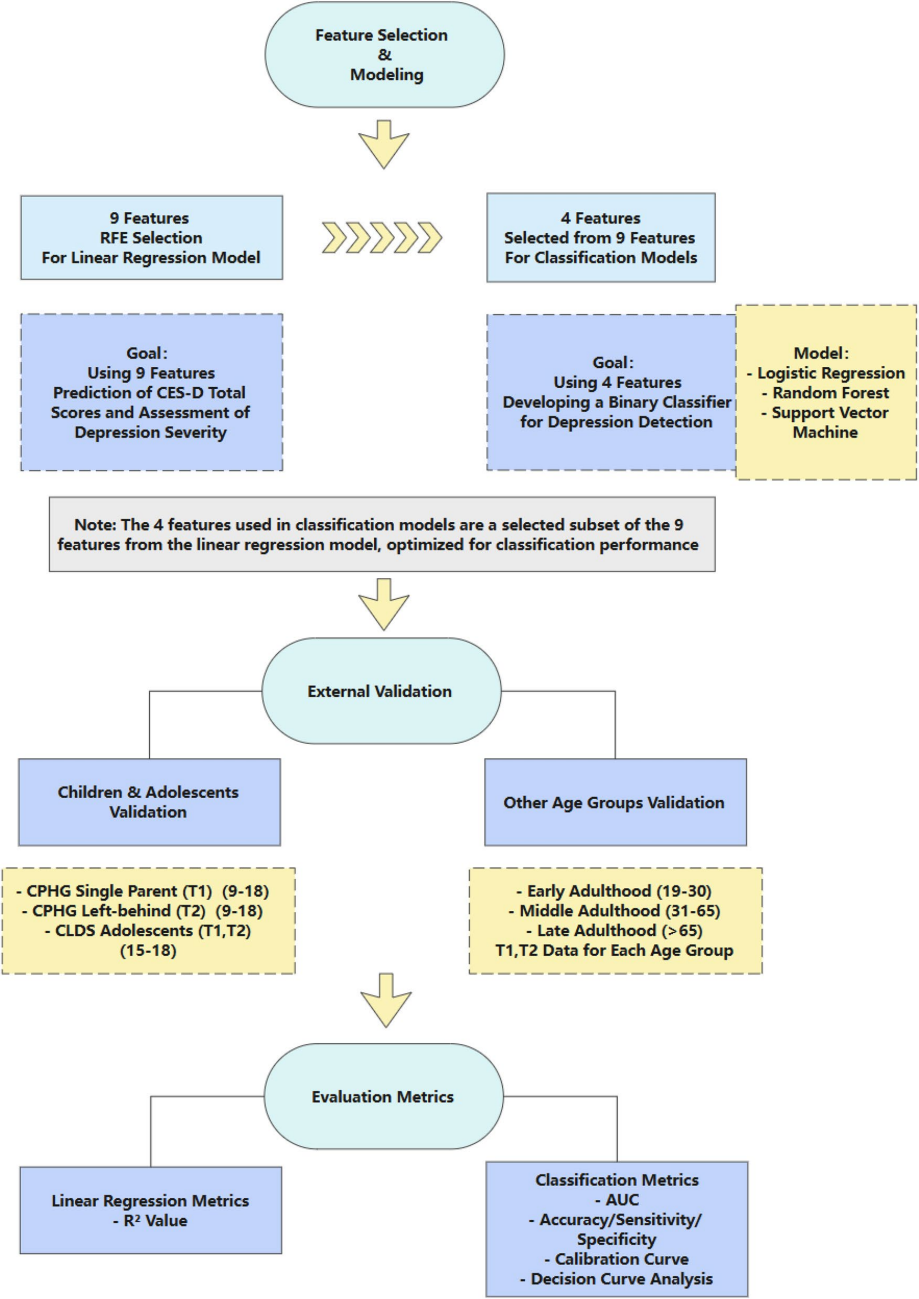
Methodological framework: systematic approach for development and validation of stratified CES-D screening system through machine learning techniques

The data analysis was conducted using Python 3.9 platform, with model training and evaluation implemented through the scikit-learn 1.5.2 machine learning library. Parallel computing techniques were incorporated to enhance computational efficiency, ensuring smooth implementation of large-scale cross-validation. The study strictly adhered to machine learning best practice guidelines, implementing effective measures to prevent data leakage and ensure the reliability and reproducibility of research findings. The research procedure is depicted in Fig. 1.

## Results

### Recursive Feature Elimination (RFE) and linear regression model

Through Recursive Feature Elimination (RFE) combined with cumulative variance explanation (R^2^) analysis, this study identified 9 core items with significant predictive power from the CES-D-20 scale. Unlike traditional scale reduction methods that set a predetermined target for the number of retained items, our approach employs Recursive Feature Elimination (RFE), a data-driven method that systematically identifies the optimal item set. The selection of 9 items was based on detecting the inflection point in the R^2^ curve, where additional items contributed minimal incremental variance explanation. This approach ensures that the retained items maximize predictive efficiency without relying on arbitrary cutoffs.

These items encompass multiple dimensions of depressive symptoms: emotional symptoms (C06 "I felt depressed", C12 "I felt unhappy", C18 "I felt sad"), cognitive symptoms (C09 "I thought my life had been a failure"), somatic symptoms (C02 "I did not feel like eating; my appetite was poor", C07 "I felt that everything I did was an effort"), interpersonal symptoms (C14 "I felt lonely", C19 "People were unfriendly"), and emotional vulnerability (C01 "I was bothered by things that usually don't bother me"). Tese results can be found in Table 1.

**Table 1:** Recursive feature elimination results for CES-D items: rankings and cumulative variance explained (R^2^) by progressive item addition, ordered by mean feature importance ranking

No	CES-D Code	Item Description	Mean ranking	Cumulative R^2^
1	C18	I feel sad	1	0.6433
2	C09	I think my life has been a failure	1.05	0.7782
3	C06	I feel depressed	1.15	0.8527
4	C19	I feel that people dislike me	1.3	0.8827
5	C12	I feel unhappy	1.5	0.9035
6	C07	I feel that everything I do is an effort	1.75	0.9231
7	C14	I feel lonely	2.05	0.9349
8	C02	I don't feel like eating; my appetite is poor	2.4	0.9473
9	C01	Some things that usually don't bother me make me feel annoyed	2.8	0.9565
10	C10	I feel fearful	3.25	0.9630
11	C16	I do not enjoy life	3.75	0.9666
12	C04	I feel I am not good as same as other people	4.3	0.9741
13	C11	My sleep is restless	4.9	0.9801
14	C05	I have trouble keeping my mind on what I'm doing	5.55	0.9864
15	C15	People are unfriendly	6.25	0.9886
16	C13	I eat less than usual	7	0.9918
17	C17	I have crying spells	7.8	0.9949
18	C08	I feel depressed about the future	8.65	0.9975
19	C03	I feel that I can't shake off the blues even with help from my family or friends	9.55	1.0000
20	C20	I walk slowly	10.5	1.0000

**Fig. 2:**
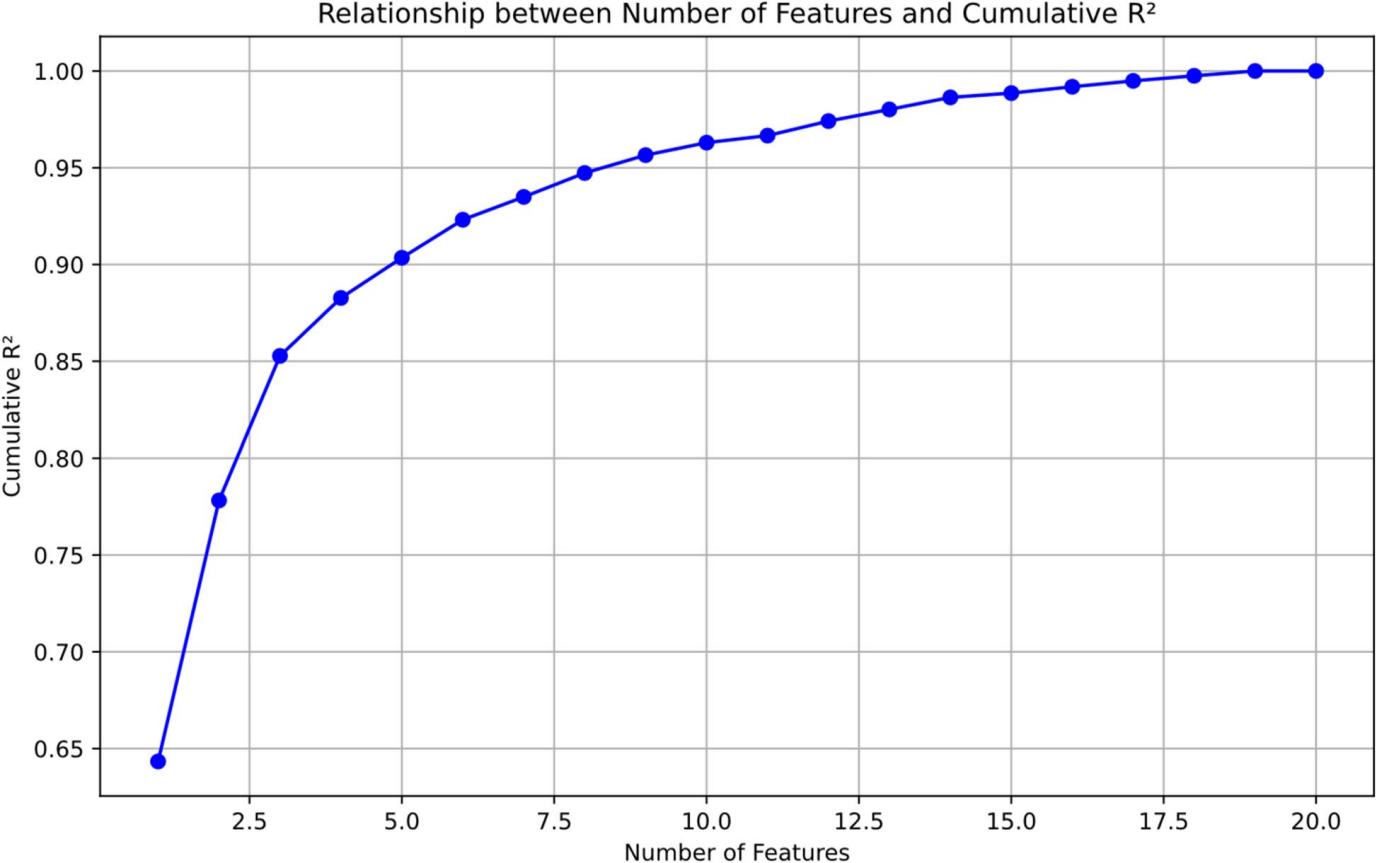
Relationship between number of features and cumulative variance explained (R.^2^): analysis of incremental predictive value with progressive feature addition. Model equation: Total = 0.6660 + 1.6584*C01 + 1.6832*C02 + 1.7542*C06 + 2.0579*C07 + 2.4517*C09 + 1.7700*C12 + 1.8957*C14 + 2.0316*C18 + 2.1980*C19

The linear regression model constructed based on these 9 core items demonstrated excellent predictive performance. In tenfold cross-validation, the model achieved an average variance explanation (R^2^) of 0.9565, indicating that this abbreviated version effectively captures the severity of depressive symptoms reflected in the original scale. The model obtained an R^2^ value of 0.9572 on the independent test set, further confirming its robust generalizability. As shown in Fig. 2, the cumulative variance explanation rate plateaus when the number of features exceeds 9, suggesting that additional items provide limited marginal information, thus supporting the feasibility of scale simplification.

The final regression equation is as follows: Total = 0.6660 + 1.6584 × C01 + 1.6832 × C02 + 1.7542 × C06 + 2.0579 × C07 + 2.4517 × C09 + 1.7700 × C12 + 1.8957 × C14 + 2.0316 × C18 + 2.1980 × C19.

Analysis of the regression coefficients reveals that C09 (β = 2.4517) contributes most substantially to the prediction of total scores, followed by C19 (β = 2.1980) and C07 (β = 2.0579), suggesting that cognitive symptoms (negative life evaluation), impaired social functioning, and somatic symptoms may be critical indicators in assessing the severity of depression among adolescents.

### Feature selection and classification model development

Through Recursive Feature Elimination analysis, we successfully identified a simplified yet efficient subset of CES-D items for predicting clinically significant depressive symptoms. The analysis revealed that among the original 20 items, only 4 core items were necessary to achieve excellent predictive performance. These four items are C18, C09, C06, and C19, encompassing key dimensions of emotional and cognitive depressive symptoms.

**Fig. 3:**
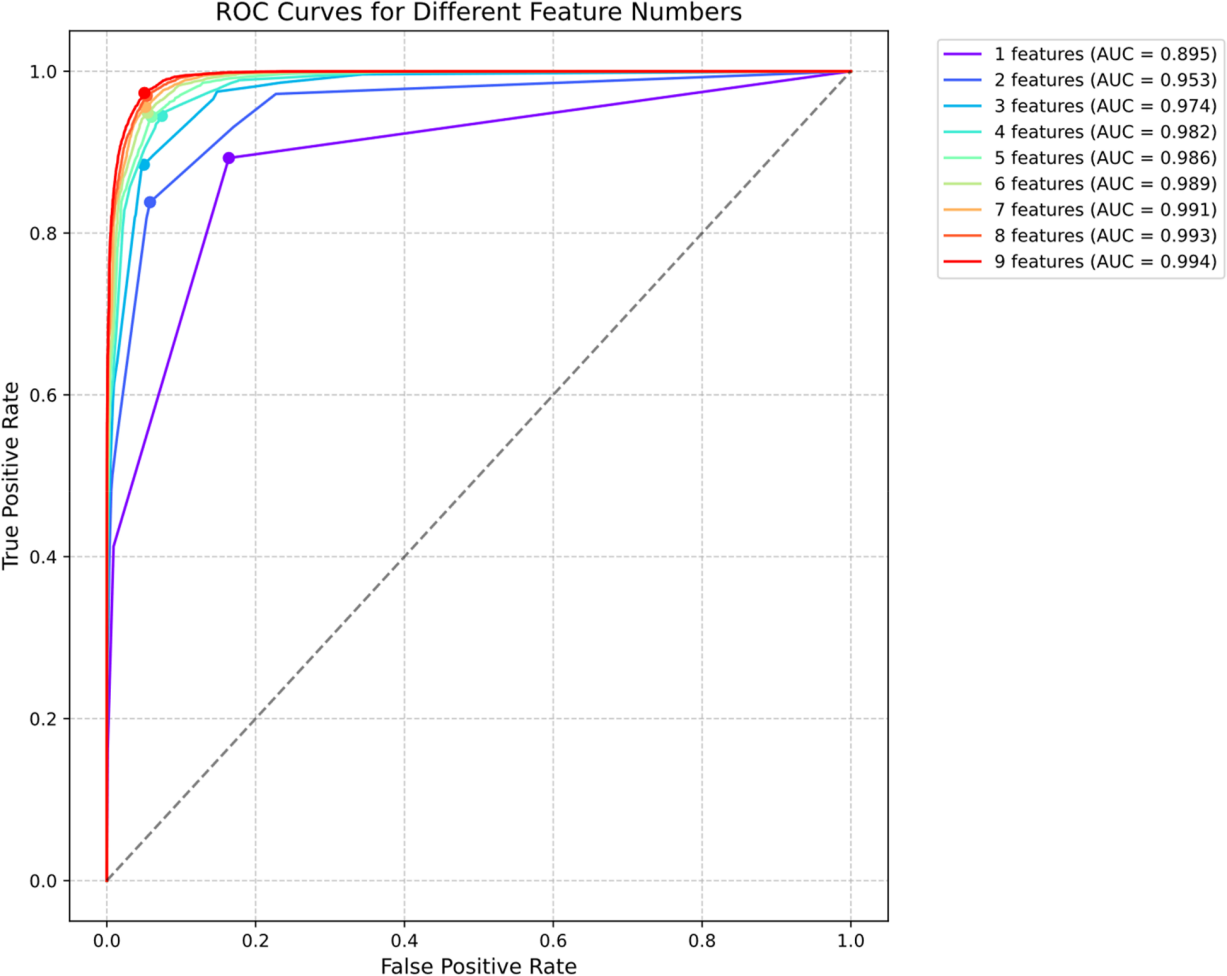
ROC curves analysis for different feature combinations: comparison of classification performance with incremental feature addition from core items

**Fig. 4:**
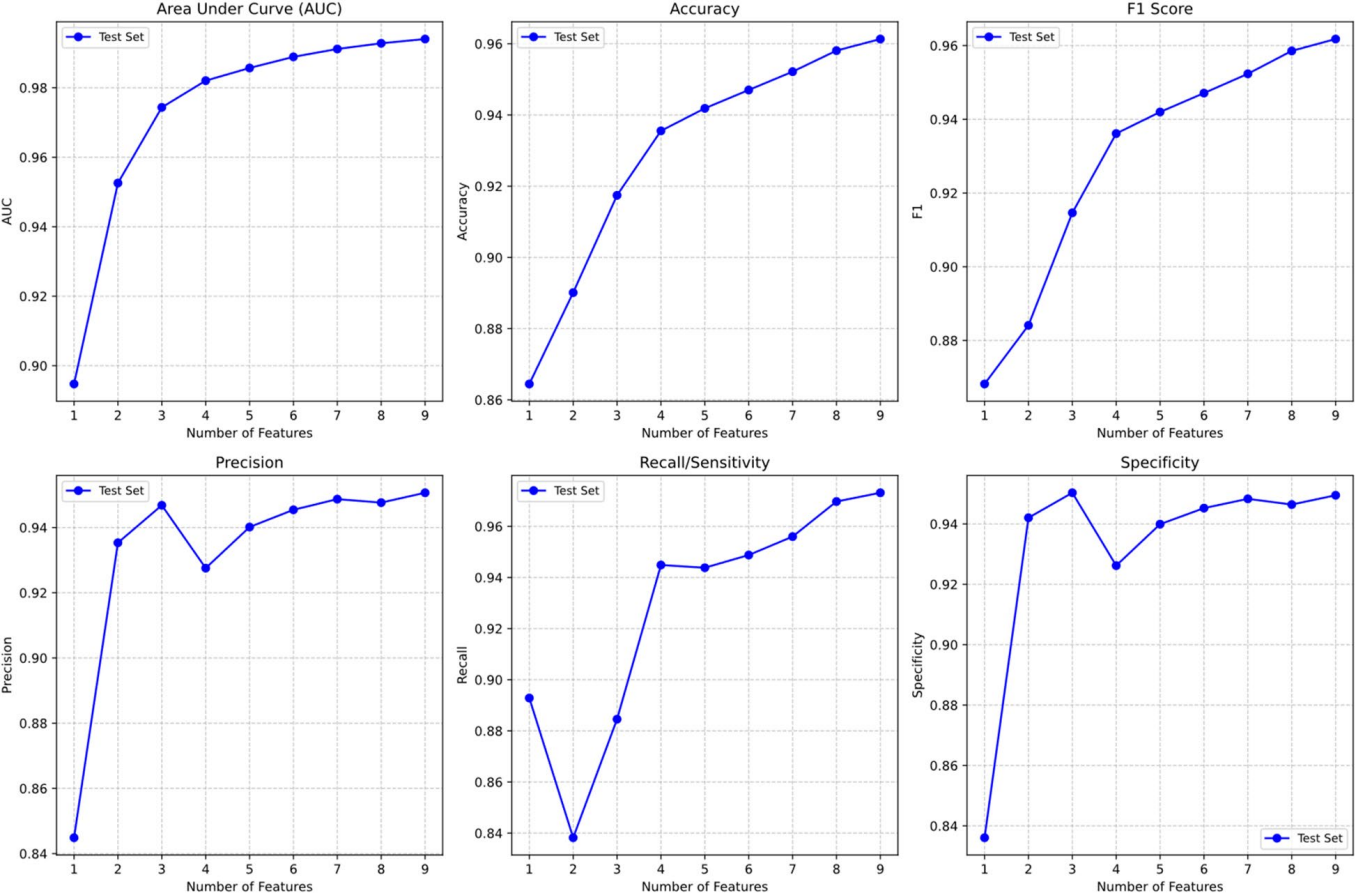
Multi-metric evaluation of model performance: changes in classification metrics (AUC, accuracy, F1 score, sensitivity, specificity, precision) with increasing item numbers

The logistic regression model constructed based on these four core items demonstrated outstanding diagnostic performance, achieving an Area Under the ROC Curve (AUC) of 0.98. Using the Youden index, the optimal classification threshold was determined to be 0.5808. At this cutoff point, the model exhibited exceptional diagnostic metrics across all parameters: sensitivity of 0.9449, indicating accurate identification of 94.49% of individuals with clinically significant depressive symptoms; specificity of 0.9262, suggesting correct screening of 92.62% of individuals without significant depressive symptoms. The model achieved an F1 score of 0.9361, reflecting an excellent balance between precision and recall, with an overall accuracy of 93.55%, highlighting its robust classification capabilities. Tese results can be found in Table 2. The AUC values and various diagnostic metrics under different feature combinations are shown in Figs. 3 and 4.

**Table 2:** Classification performance metrics for cumulative item combinations (*n* = 179,877)

Feature size	AUC	Accuracy	F1	Sensitivity	Specificity	Precision
1	0.8947	0.8645	0.8682	0.8928	0.8361	0.8449
2	0.9526	0.8901	0.8841	0.8382	0.9420	0.9353
3	0.9743	0.9174	0.9146	0.8846	0.9503	0.9468
4	0.9820	0.9355	0.9361	0.9449	0.9262	0.9276
5	0.9857	0.9419	0.9420	0.9438	0.9399	0.9402
6	0.9889	0.9470	0.9471	0.9488	0.9452	0.9454
7	0.9911	0.9522	0.9523	0.9560	0.9483	0.9487
8	0.9928	0.9581	0.9586	0.9697	0.9464	0.9476
9	0.9940	0.9613	0.9618	0.9732	0.9495	0.9507

1: C18

2: C18, C09

3: C18, C09, C06

4: C18, C09, C06, C19

5: C18, C09, C06, C19, C12

6: C18, C09, C06, C19, C12, C07

7: C18, C09, C06, C19, C12, C07, C14

8: C18, C09, C06, C19, C12, C07, C14, C02

9: C18, C09, C06, C19, C12, C07, C14, C02, C01

The final logistic regression prediction equation is:

$$\text{logit}(\text{p}) = -4.6836+1.7763*\text{C}18 + 1.7766*\text{C}09 + 1.9443*\text{C}06 + 1.6167*\text{C}19$$

Here, *p* represents the probability of an individual's CESD total score being ≥ 16, while C18, C09, C06, and C19 represent the original scores (0–3 points) for their respective items. The regression coefficients indicate comparable predictive contributions from these items, underscoring their balanced importance in the assessment process.

### Comparative analysis of multiple classification models

**Fig. 5:**
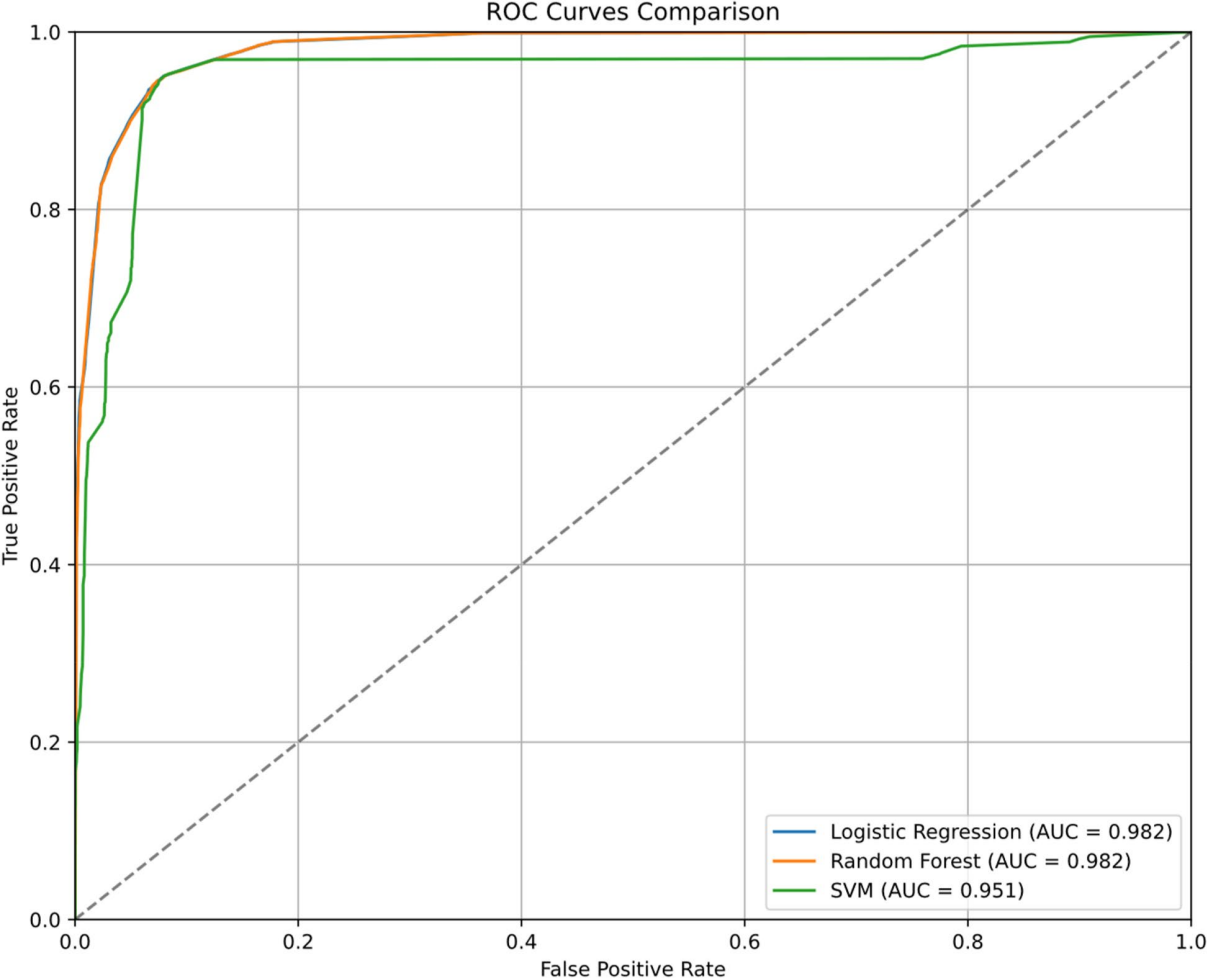
Comparative ROC curves analysis: performance comparison of logistic regression, random forest, and support vector machine models using four core items

To determine the optimal classification algorithm, we conducted rigorous statistical comparisons among three machine learning models. One-way Analysis of Variance (ANOVA) revealed significant performance differences between models (F = 267.1417, *p* < 0.001). Subsequent Tukey HSD post-hoc tests demonstrated comparable performance levels between logistic regression and random forest models (*p* = 0.9996, 95% CI containing 0), while both significantly outperformed the support vector machine model (*p* < 0.001). These findings provided robust statistical evidence for model selection. For detailed comparison results, please refer to Fig. 5 and Table 3.

**Table 3:** Comparative analysis of classification performance across three machine learning algorithms using four core items

Dataset	Logistic Regression	Random Forest	SVM
AUC	0.9804	0.9804	0.9554
Accuracy	0.9355	0.9355	0.9354
Sensitivity (Recall)	0.9449	0.9449	0.9504
Specificity	0.9262	0.9262	0.9203
Precision	0.9276	0.9276	0.9226
F1 Score	0.9361	0.9361	0.9363

Between the two models with comparable performance, logistic regression demonstrated distinct advantages. Primarily, as a linear model, it exhibits low computational complexity, particularly suitable for prediction tasks with fewer features. More importantly, logistic regression offers excellent interpretability, allowing for intuitive quantification of feature contributions to predictions through regression coefficients. This transparency holds particular significance in mental health screening applications, enabling clinicians to understand and interpret the sources of predictive outcomes.

In contrast, while the random forest model can capture non-linear relationships between features, its "black box" nature limits the intuitive interpretation of the model.

Based on this comprehensive analysis, we selected logistic regression as the final predictive model. This choice was driven not only by its superior predictive performance but, crucially, by its outstanding interpretability and practicality—characteristics that hold substantial value in developing a simplified yet effective screening tool for depressive symptoms.

### External validation

#### Children and adolescents group validation

To assess the model's generalization performance in child and adolescent populations, we conducted rigorous external validation using four independent samples. Specifically, we first identified nine most influential features through Recursive Feature Elimination (RFE) from the original dataset, then extracted these features from new datasets. During data preprocessing, we calculated total scores and eliminated instances with missing values to ensure data completeness. Subsequently, we applied the fitted linear regression model for prediction and comprehensively examined its generalization capability through multiple evaluation metrics. In terms of explanatory power, the model demonstrated excellent performance across all validation samples of children and adolescents. The R^2^ reached 0.957 for CPHG single-parent family samples (*n* = 48,128), 0.962 for CPHG left-behind children T2 samples (*n* = 133,904), 0.937 for CLDS adolescent T1 samples (*n* = 1,075), and 0.947 for T2 samples (*n* = 742) (Table 4).

**Table 4:** Cross-sample validation of nine-item linear regression model

Dataset	R^2^
CPHG_SPCA_T1	0.9569
CPHG_LBCA_T2	0.9621
CLDS_CA_T1	0.9365
CLDS_CA_T2	0.9473

**Fig. 6:**
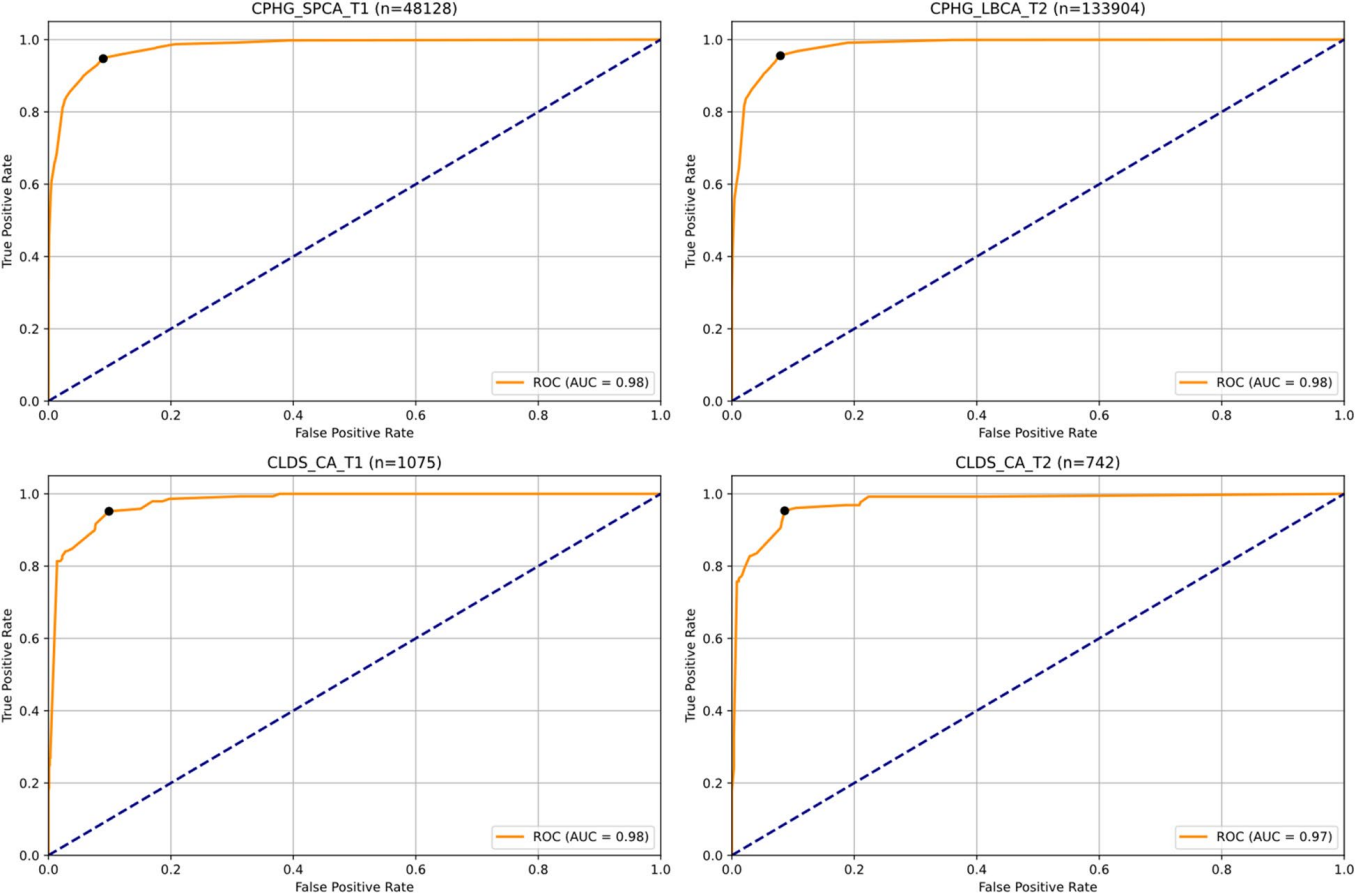
Cross-sample validation of four-item classification model: ROC curves analysis across different databases and time points

To validate the four-feature classification model, we extracted these features from new datasets and applied the fitted logistic regression model for classification. The results demonstrated excellent classification performance across all samples (Table 5, Fig. 6). The CPHG single-parent family sample showed outstanding performance with an accuracy of 0.918, sensitivity and specificity of 0.947 and 0.911 respectively, F1 score of 0.825, and an AUC of 0.979. Similarly, the CPHG left-behind children T2 sample exhibited remarkable results with an accuracy of 0.928, sensitivity and specificity of 0.955 and 0.921 respectively, F1 score of 0.838, and an AUC of 0.983. The CLDS database also yielded reliable validation results across two time points: T1 sample (*n* = 1,075) achieved an accuracy of 0.908, F1 score of 0.736, and AUC of 0.977; T2 sample (*n* = 742) showed an accuracy of 0.921, F1 score of 0.805, and AUC of 0.975. Notably, sensitivity remained above 0.95 across all samples, indicating stable depression risk identification capability. The consistently high accuracy rates above 0.90 across all samples further confirmed the model's robust classification performance in child and adolescent populations.

**Table 5:** External validation results of four-item classification model

Dataset	n	AUC	Accuracy	F1	Sensitivity	Specificity	Precision
CPHG_SPCA_T1	48,128	0.9791	0.9180	0.8251	0.9472	0.9105	0.7309
CPHG_LBCA_T2	133,904	0.9825	0.9275	0.8378	0.9553	0.9207	0.7461
CLDS_CA_T1	1,075	0.9772	0.9079	0.7360	0.9517	0.9011	0.6000
CLDS_CA_T2	742	0.9750	0.9205	0.8053	0.9531	0.9137	0.6971

**Fig. 7:**
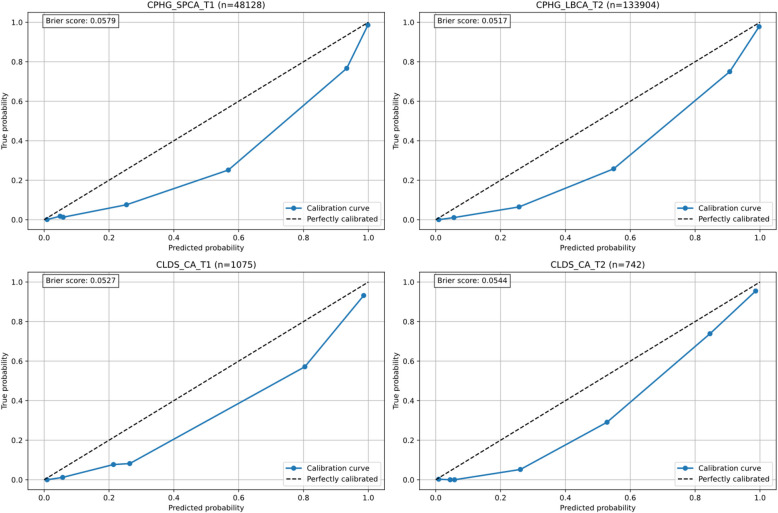
Calibration plot analysis: assessment of predicted probability accuracy across different samples and time points

The model's predictive probability calibration was evaluated through calibration curves (Fig. 7). The simplified scale demonstrated good calibration performance across multiple child and adolescent samples. Specifically, in the CPHG single-parent children (CPHG_SPCA_T1) sample, the calibration curve closely approximated the ideal 45-degree diagonal line, with a Brier score of 0.0579, indicating high consistency between predicted probabilities and actual observed outcomes. In the CPHG left-behind children follow-up sample (CPHG_LBCA_T2), the model also exhibited excellent calibration performance, with a Brier score of 0.0517 and high concordance between the calibration curve and diagonal line.

Analysis of the CLDS adolescent samples further validated the model's robustness. Despite the relatively small sample size (*n* = 1,075) at baseline measurement (CLDS_CA_T1), the calibration curve showed good fit with a Brier score of 0.0527. At the two-year follow-up measurement (CLDS_CA_T2), the model maintained stable calibration performance with a Brier score of 0.0544. Notably, across all samples, the calibration curves demonstrated optimal fitting in the medium to high-risk range, which precisely covers the most critical risk assessment interval in clinical decision-making.

The calibration curve analysis results indicate that the simplified scale, constructed from four core items, not only possesses good discriminative ability but also accurately estimates individual depression risk probabilities, which is crucial for risk assessment and intervention decisions in clinical practice. The low Brier scores (all < 0.06) further confirm the reliability of the model's predictions, supporting the practical value of this simplified scale as a screening tool for adolescent depressive symptoms.

**Fig. 8:**
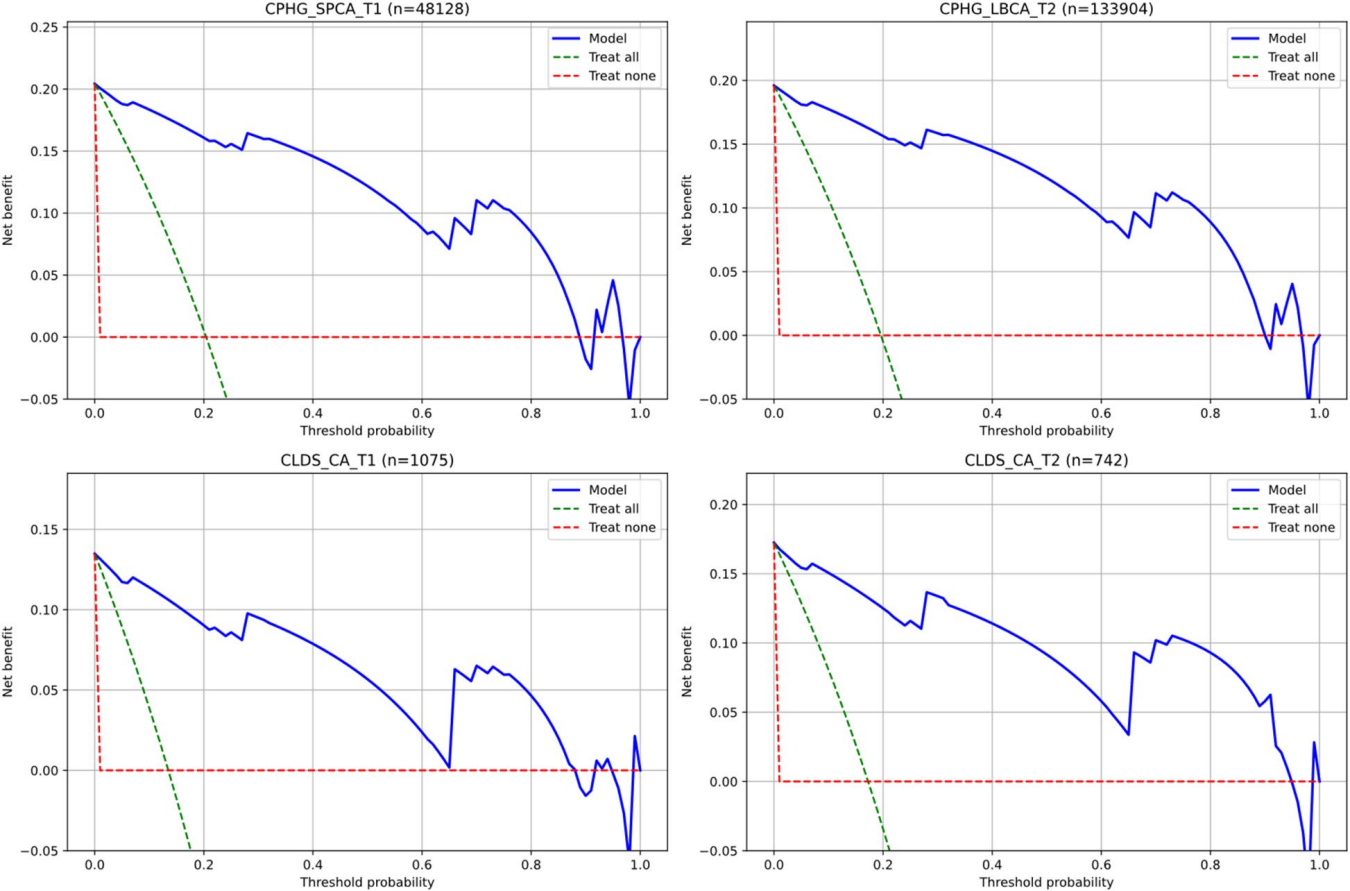
Decision curve analysis: net benefit assessment of the screening system across different risk thresholds in various population samples

In the clinical application assessment for child and adolescent populations, decision curve analysis revealed significant screening value of the simplified CESD scale, as shown in Fig. 8. Specifically, in two large-scale populations—the CPHG single-parent children (*n* = 48,128) and left-behind children follow-up samples (*n* = 133,904)—the model demonstrated excellent and consistent net benefit performance. When risk thresholds ranged between 0.2 and 0.8, the model's decision curves significantly outperformed both extreme strategies of "treat all" and "treat none," providing superior options for clinical decision-making.

Analysis of the CLDS adolescent samples further strengthened these findings. In both baseline (*n* = 1,075) and two-year follow-up (*n* = 742) data, despite relatively smaller sample sizes, the model maintained stable advantages in decision curves. Notably, the model showed maximum net benefit in the clinically critical decision interval of 0.3–0.6, a characteristic particularly significant for balancing screening sensitivity and specificity.

Particularly noteworthy is the model's demonstration of robust decision curve characteristics across child and adolescent populations of varying sizes and backgrounds. This cross-sample consistency highlights its universality as a depression symptom screening tool. The stable trajectory of net benefit curves indicates that this simplified scale can provide reliable guidance for clinicians' decisions across different risk thresholds, effectively enhancing early identification efficiency of depressive symptoms.

#### Cross-age group validation

To comprehensively assess the model's applicability across different age groups, this study employed longitudinal data from various age cohorts within the CLDS database for validation. The validation sample encompassed three age groups: early adulthood (19–30 years), middle adulthood (31–65 years), and late adulthood (> 65 years). For each age group, data included baseline measurements from 2016 (T1) and follow-up measurements from 2018 (T2).

Regarding the linear regression models, the nine core features demonstrated exceptional explanatory power across all age groups in the validation samples. As shown in Table 6, the variance explained (R^2^) for the early adulthood group reached 0.951 and 0.950 in the baseline and follow-up samples, respectively. The middle adulthood group maintained stable R^2^ values of 0.952 and 0.951, while the late adulthood group showed R^2^ values of 0.952 and 0.947. These findings indicate that the simplified scale exhibits significant and consistent effectiveness in explaining depressive symptom variance across all age groups, with R^2^ values consistently maintaining high levels above 0.94 across all samples.

**Table 6:** Age-stratified validation of nine-item linear regression model

Dataset	R^2^
CLDS_A_T1(2016)	0.9512
CLDS_B_T1(2016)	0.9515
CLDS_C_T1(2016)	0.9523
CLDS_A_T2(2018)	0.9505
CLDS_B_T2(2018)	0.9515
CLDS_C_T2(2018)	0.9475

A denotes early adulthood (19–30 years), B denotes middle adulthood (31–65 years), and C denotes late adulthood (> 65 years)

**Fig. 9:**
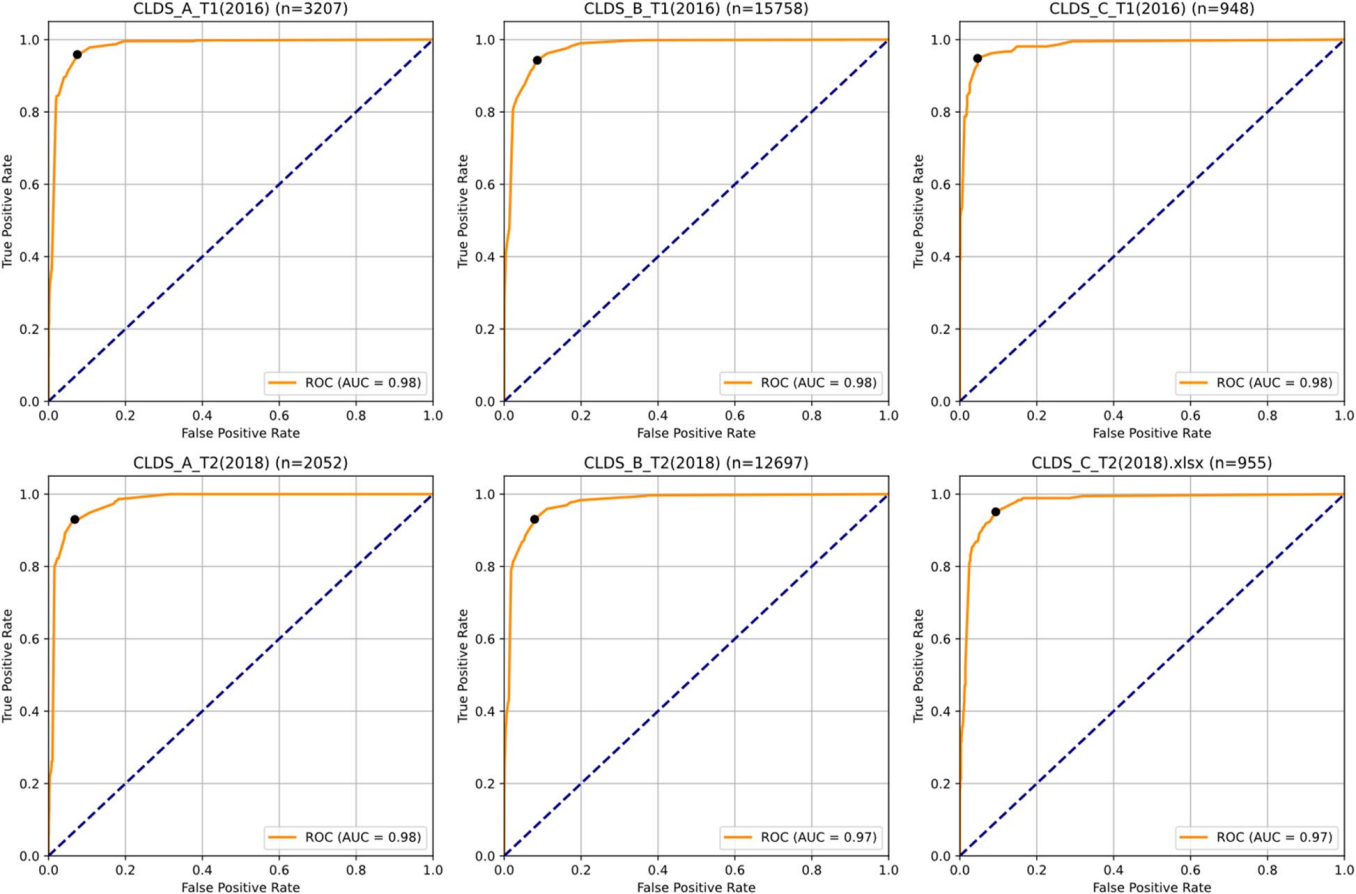
Age-stratified ROC curves analysis: classification performance of four-item model across different age groups and time points

As shown in Table 7 and Fig. 9, in validating the four-feature classification model, all age groups demonstrated excellent and stable predictive performance. The early adulthood sample (CLDS_A) performed exceptionally well in both baseline (*n* = 3,207) and follow-up (*n* = 2,052) measurements, achieving AUC values of 0.980 and 0.977, respectively, with accuracy maintained around 0.93, and both sensitivity and specificity remaining above 0.93. The middle adulthood sample (CLDS_B), which had the largest sample size (baseline *n* = 15,758; follow-up *n* = 12,697), also showed satisfactory validation results, with AUC values of 0.976 and 0.975 for baseline and follow-up measurements, accuracy maintained around 0.92, and both sensitivity and specificity exceeding 0.91. Although the late adulthood sample (CLDS_C) had a relatively smaller sample size (baseline *n* = 948; follow-up *n* = 955), the model performance remained robust, with AUC values of 0.983 and 0.975 for baseline and follow-up, achieving accuracy rates of 0.953 and 0.915, respectively.

**Table 7:** Age-stratified external validation of four-item classification model

Dataset	n	AUC	Accuracy	F1	Sensitivity	Specificity	Precision
CLDS_A_T1(2016)	3,207	0.9804	0.9298	0.7964	0.9586	0.9250	0.6811
CLDS_B_T1(2016)	15,758	0.9764	0.9190	0.8064	0.9429	0.9138	0.7044
CLDS_C_T1(2016)	948	0.9828	0.9525	0.8993	0.9481	0.9538	0.8553
CLDS_A_T2(2018)	2,052	0.9768	0.9313	0.8303	0.9299	0.9316	0.7500
CLDS_B_T2(2018)	12,697	0.9746	0.9227	0.8206	0.9303	0.9210	0.7340
CLDS_C_T2(2018)	955	0.9746	0.9152	0.8121	0.9511	0.9066	0.7085

Cross-age group comparative analysis revealed that both linear regression and classification models maintained high predictive accuracy across all age groups. Linear regression results showed minimal variation in R^2^ values across age groups (range: 0.947–0.952), indicating model stability in explaining depressive symptoms across age spans. Regarding classification performance, accuracy rates were maintained between 0.915–0.953 across all samples, demonstrating excellent risk identification capability (sensitivity 0.930–0.959). Notably, the late adulthood sample showed the most outstanding overall performance in the 2016 baseline measurement, achieving a precision of 0.855 and an F1 score of 0.899, suggesting particularly ideal predictive capability in the elderly population. While the middle adulthood sample had the largest sample size, its stable performance across all indicators demonstrated the model's reliability in large-sample settings. The early adulthood sample maintained high predictive performance at both time points, with the follow-up sample showing an improved F1 score (0.830) compared to baseline (0.796), indicating stable or even enhanced predictive capability over time.

**Fig. 10:**
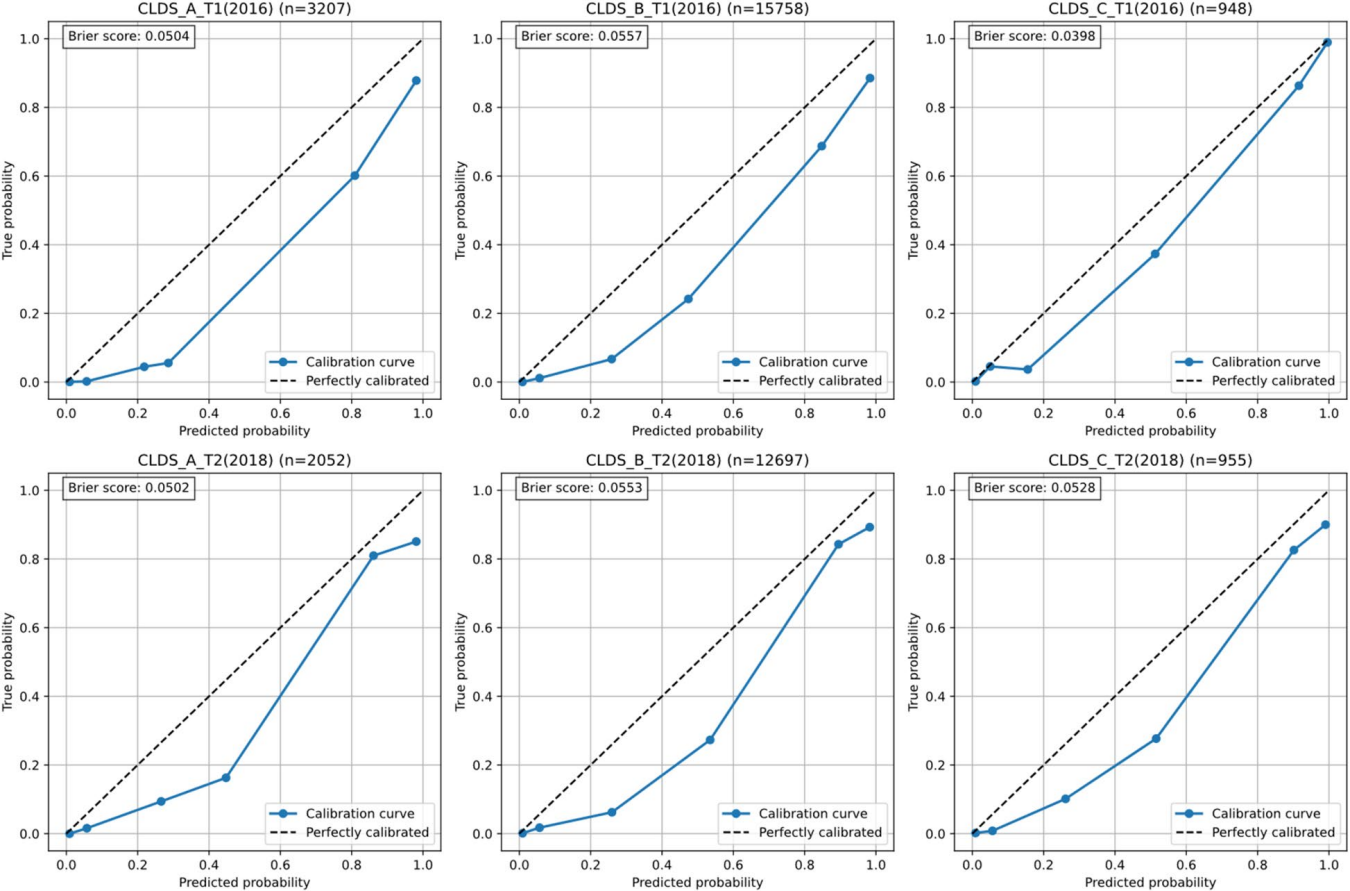
Age-stratified calibration analysis: assessment of predicted probability accuracy across different age groups and time points

The model's predictive accuracy across different age groups and time points was evaluated through calibration curves and Brier scores (Fig. 10). Results demonstrated good and stable calibration performance across all six validation datasets. Brier scores ranged from 0.0398 to 0.0557, indicating excellent predictive accuracy (all scores < 0.10). Specifically, in the 2016 datasets, CLDS_C_T1 (*n* = 948) exhibited the best calibration performance with a Brier score of 0.0398, followed by CLDS_A_T1 (*n* = 3,207) with 0.0504 and CLDS_B_T1 (*n* = 15,758) with 0.0557. The 2018 datasets showed similar patterns, with Brier scores of 0.0502 for CLDS_A_T2 (*n* = 2,052), 0.0553 for CLDS_B_T2 (*n* = 12,697), and 0.0528 for CLDS_C_T2 (*n* = 955).

The calibration analyses specifically addressed concerns about potential score inflation across different response patterns. The close alignment between predicted probabilities and observed outcomes (Brier scores ranging from 0.0398 to 0.0579) indicates that the simplified versions maintain accuracy regardless of whether participants scored highly on the selected items or showed different response patterns. This robustness is further supported by the consistent performance across diverse validation samples, suggesting that the models effectively capture depression severity without systematic bias toward particular response patterns.

Calibration curves consistently demonstrated a slight tendency toward risk overestimation, particularly in the low probability range (0–0.4), while showing better calibration performance in the high probability range (0.8–1.0). This systematic pattern remained consistent across different age groups and time points, indicating stable model performance. The findings suggest that the model performs exceptionally well in identifying high-risk individuals, although it slightly tends to overestimate actual risk in low-risk populations.

**Fig. 11:**
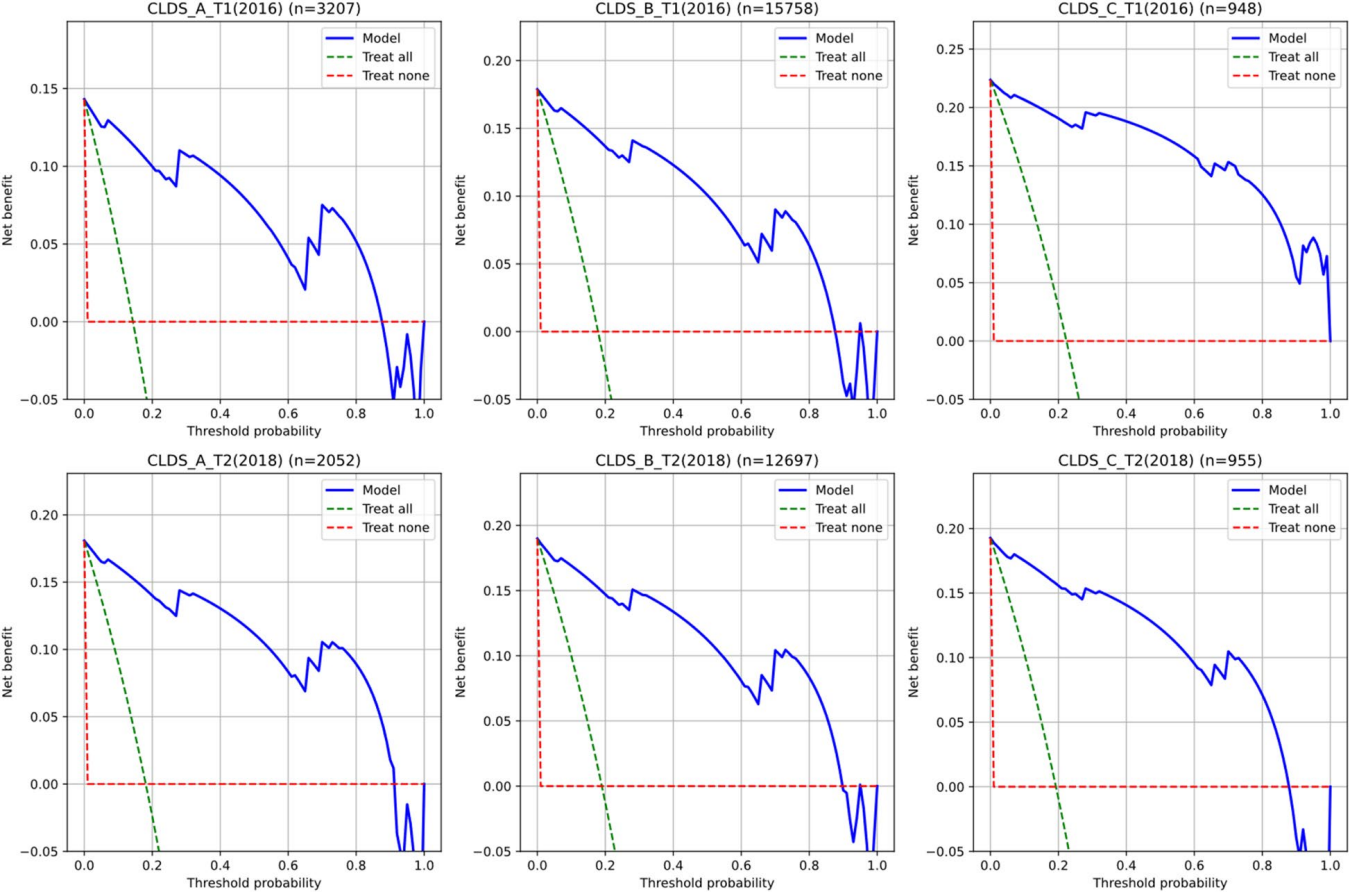
Age-stratified decision curve analysis: net benefit assessment across different risk thresholds for various age groups

Decision Curve Analysis (DCA) was conducted on six independent validation datasets to further evaluate the clinical utility of the simplified CESD scale (Fig. 11). Results demonstrated significant clinical net benefit of the four-feature logistic regression model across all validation samples. Specifically, in the 2016 baseline data, the decision curves for early adulthood (CLDS_A_T1), middle adulthood (CLDS_B_T1), and late adulthood (CLDS_C_T1) samples outperformed both extreme strategies of "treat all" and "treat none" across most threshold probabilities (approximately 0.2–0.8). The model maintained stable and substantial net benefits within the critical decision threshold range of 0.2–0.8. The 2018 follow-up data validation results (CLDS_A_T2; CLDS_B_T2; CLDS_C_T2) exhibited similar patterns, indicating good temporal stability in the model's clinical value.

Notably, the model demonstrated maximum net benefit within the threshold probability range of 0.3–0.6, which precisely corresponds to the most commonly used decision threshold range in clinical practice. This finding corroborates the previous calibration curve analysis results, further supporting the simplified scale's advantages in identifying moderate to high-risk individuals. Cross-age group comparisons revealed comparable decision benefits across all age groups despite substantial differences in sample sizes, highlighting the model's broad applicability. These results align with the model's excellent performance in discrimination (AUC) and calibration (Brier scores), collectively confirming the practical value of the simplified CESD scale as a depression symptom screening tool.

## Discussion

### Summary of key findings and innovation

This study advances depression screening methodology through innovative applications of machine learning techniques. Our investigation systematically quantified the relationship between CES-D item quantity and predictive power, revealing that just nine items can capture 95.65% of the variance in total depression scores, while four items achieve exceptional classification accuracy (AUC = 0.982) for identifying clinically significant depressive symptoms. The robustness of these findings is demonstrated through extensive validation across diverse populations and age groups, using a large primary sample (*n* = 179,877) and multiple independent validation cohorts. The regression model maintained R^2^ values above 0.94 across all age groups, while the classification model consistently achieved AUC values above 0.97. Calibration analyses showed excellent concordance between predicted probabilities and observed outcomes (Brier scores ranging from 0.0398 to 0.0579), and decision curve analyses revealed substantial net clinical benefit across a wide range of threshold probabilities (0.2–0.8), particularly in the clinically critical decision interval of 0.3–0.6.

### Methodological breakthroughs and theoretical contributions

The integration of Recursive Feature Elimination (RFE) with comprehensive validation in our study represents both methodological innovation and theoretical advancement in psychometric assessment. Unlike traditional factor analytic approaches that primarily focus on internal structure, our method provides the precise quantification of each item's predictive contribution to depression assessment. This innovation enables evidence-based decisions about item retention based on quantifiable predictive value rather than conventional arbitrary thresholds [17, 18]. To ensure the generalizability of the selected items, we implemented a multi-sample external validation strategy across independent datasets spanning different age groups and socioeconomic backgrounds. The consistent high predictive performance of the selected items (R^2^ > 0.94, AUC > 0.97) suggests that the feature selection process did not merely favor commonly endorsed items within a single population but identified items with stable predictive value across diverse demographic contexts. Additionally, the overlap between our selected items and prior validated short versions of CES-D reinforces their psychometric robustness.The method's ability to identify optimal feature combinations while accounting for complex item interactions transcends the limitations of classical test theory approaches, which often treat items as independent contributors [19, 20].

Our findings establish a mathematical model describing the non-linear relationship between item quantity and predictive power, demonstrating rapid initial gains that plateau around nine items. This quantitative framework challenges the traditional assumption that more items inherently lead to better validity, providing empirical evidence that carefully selected core items can capture the essential aspects of depression measurement [21]. The high predictive accuracy achieved with minimal items supports recent theoretical frameworks suggesting that depression manifests through a hierarchical network of symptoms rather than as a uniform construct [22, 23].

The success of our data-driven progressive screening framework provides strong empirical support for the hierarchical structure theory of depressive symptoms. The high predictive power of specific items related to mood (C18 "I feel sad", C06 "I feel depressed"), cognitive symptoms (C09 "I think my life has been a failure"), and social functioning (C19 "I feel that people dislike me") aligns with theoretical models emphasizing the centrality of these domains in depression manifestation [24, 25]. These four core items represent distinct but interconnected pathways in the depression network: emotional dysregulation (C18, C06), negative cognitive schemas (C09), and social disconnection (C19). Recent network analyses have demonstrated similar interconnected relationships among emotion regulation difficulties, anxiety, and depression in adolescents [26, 27]. The remarkable predictive accuracy achieved by this minimal item set suggests that these symptoms may serve as central nodes in the depression network, triggering and maintaining other symptoms through complex feedback loops [28, 29]. This finding contributes to the growing evidence that depression may be better understood as a network of interconnected symptoms rather than a unitary construct, where specific symptom clusters can effectively capture the overall severity of the condition.

Most significantly, our methodology bridges the theoretical gap between rapid screening and comprehensive assessment by introducing a dynamic, integrated approach to depression evaluation. The demonstrated stability of both brief (4-item) and extended (9-item) models across diverse populations suggests that depression screening can be conceptualized as a continuous process rather than a binary choice [30, 31]. This integration challenges traditional dichotomies in psychological assessment, offering a new theoretical model where assessment depth can be dynamically adjusted based on initial responses [32]. Through systematic evaluation of cumulative item contributions, our framework enables sophisticated staging algorithms where specific response patterns to the four core items can trigger targeted administration of additional items, creating personalized assessment pathways. This machine learning-supported adaptive approach optimizes both efficiency and diagnostic accuracy, demonstrating how computational methods can enhance traditional psychological assessment paradigms [33, 34].

### Dialogue with previous CES-D simplification studies

While earlier CES-D simplification studies made important contributions, our study provides complementary evidence through different methodological approaches. Unlike Baron et al.'s [6] 10-item version and Van de Velde et al.'s [5] 8-item version which used traditional psychometric methods, our machine learning approach through Recursive Feature Elimination enables precise quantification of each item's predictive contribution to depression assessment.

The performance comparison with previous shortened versions is particularly noteworthy. Baron et al. [6] reported optimal cut-offs varying by language groups (Zulu: sensitivity = 71.4%, specificity = 72.6%,Afrikaans: sensitivity = 84.6%, specificity = 84.0%; Xhosa: sensitivity = 81.0%, specificity = 95.0%) with AUC values ranging from 0.81 to 0.94. In comparison, our four-item screening layer achieved superior performance (sensitivity = 94.5%, specificity = 92.6%, AUC = 0.982) while using fewer items. More importantly, our nine-item comprehensive assessment maintains an exceptional explanatory power (R^2^ = 0.957) for the full scale score, compared to the relatively lower internal consistency (α = 0.69–0.89) reported in Baron's study.

This improved performance is validated through a substantially larger sample (*N* = 179,877) compared to previous studies (e.g., Baron et al.'s *N* = 944), along with comprehensive examination of calibration (Brier scores ranging from 0.0398 to 0.0579) and clinical utility across risk thresholds. The stratified design offers both measurement precision and practical efficiency, making it particularly valuable for large-scale mental health screening programs where resource optimization is crucial.

### Clinical value and implementation efficiency

Our dual-level screening framework offers unique value in addressing the persistent challenge of balancing assessment thoroughness with clinical efficiency. The hierarchical structure allows clinicians to begin with four core items for highly reliable initial risk assessment (sensitivity > 0.93, specificity > 0.91), and seamlessly transition to the nine-item comprehensive assessment when warranted, without redundancy or loss of information. Regarding potential concerns about score inflation for participants endorsing specific items more frequently, our calibration analyses (Figs. 7 and 10) demonstrated excellent concordance between predicted probabilities and observed outcomes, with Brier scores consistently below 0.06. The decision curve analyses (Figs. 8 and 11) further confirmed that the stratified screening models provide stable net benefit across diverse risk thresholds, particularly in the clinically critical range of 0.3–0.6. These findings suggest that the predictive models effectively capture depression severity without systematic bias toward particular response patterns.

The economic implications are particularly noteworthy. By reducing the average screening time from approximately 6–8 min (full CES-D) to 2–3 min (4-item screening) for most cases while maintaining the option for more comprehensive assessment when needed, the system could significantly reduce healthcare costs and improve resource allocation. This efficiency is especially crucial in large-scale mental health screening programs. Furthermore, the framework's ability to maintain high accuracy across different severity levels addresses a critical limitation of existing brief screens, which often perform poorly at diagnostic boundaries or with subthreshold symptoms. This adaptive capability makes the system particularly valuable in primary care settings, where rapid yet reliable depression screening is essential for effective mental health integration, especially for vulnerable populations such as adolescents [27, 35, 36].

To facilitate the practical implementation of this screening system, we have developed an online app (https://ywjcrazy.github.io/CES-D/) that demonstrates the two-stage screening framework in action. This web-based implementation allows practitioners and researchers to interact with the system directly, experiencing firsthand how the adaptive screening process works in practice.

### Limitations and future directions

Our depression screening study faces several key limitations, each pointing to specific directions for future research. A limitation of our analysis is the lack of examination of non-linear relationships between items and depression outcomes using methods like SHAP analysis. Future studies could apply SHAP dependence plots to explore non-linear interactions. However, our comparison of logistic regression and random forests shows that the linear model effectively captures the relationships in this dataset. Another limitation is the predominant focus on Chinese participants in our sample population. While this provided substantial data within one cultural context, it necessitates expanded validation efforts across Western and developing nations. Future studies should conduct systematic testing with diverse cultural populations, paying particular attention to measurement invariance to ensure our assessment tools are culturally appropriate and maintain consistency across different demographic groups and societies. Additionally, our temporal validation is currently limited to only 3–6 months of observation. To address this, future research must conduct multi-year longitudinal studies to properly establish the long-term stability of our assessment system. Finally, our clinical diagnostic validation requires strengthening. Future research should focus on comparing our results with structured clinical interviews, validating our findings against established DSM-5/ICD-11 criteria, and assessing how well our system can differentiate depression from other mental health conditions. These steps are crucial for establishing the clinical utility and reliability of our assessment system.

## Conclusion

This study presents a machine learning-derived stratified screening system for the CES-D scale that offers new possibilities for efficient and accurate depression assessment. The two-tier framework, combining a four-item rapid screening with a nine-item comprehensive assessment, demonstrates exceptional diagnostic accuracy and generalizability across diverse populations while substantially reducing assessment burden.Our findings show potential for advancing psychological assessment methodology, as the machine learning approach provides a data-driven pathway for understanding symptom networks and their predictive relationships. The practical applications of this stratified screening system show promise in enhancing mental health screening efficiency, particularly in settings with limited resources.The methodological framework developed in this study offers opportunities for optimizing other psychological assessment tools through machine learning techniques. While validation across broader cultural contexts and longer time spans remains important, this approach shows considerable potential for advancing mental health screening practices, especially valuable in meeting the growing demands for accessible and efficient mental health assessment.

## Supplementary Information

Supplementary Material 1.

## Data Availability

The data that support the findings of this study were obtained from publicly available databases. The datasets can be accessed at 10.57760/sciencedb.12150 and 10.1177/2397200917735796 .
