# Supplementary material for: Machine learning-driven development of a stratified CES-D screening system: optimizing depression assessment through adaptive item selection
Source: BMC Psychiatry. 2025 Mar 26;25:286. doi: 10.1186/s12888-025-06693-8 (PMC11938587; doi:10.1186/s12888-025-06693-8)
Supplement: Supplementary file 1 — Supplementary Material 1. [file 12888_2025_6693_MOESM1_ESM.zip › Supplementary Information/Figure_6.html]

CES-D Screening System Data Flow


# Machine Learning-Driven Development of a Stratified CES-D Screening System

Data Sources

**Primary Database**  
Chinese Psychological Health Guard Project (CPHG)  
Left-behind children T1  
n = 179,877

**Validation Database 1**  
CPHG Single-parent children T1  
n = 48,128

**Validation Database 2**  
CPHG Left-behind children T2  
n = 133,904

**Age Validation (CLDS)**  
Adolescents (15-18)  
T1: n = 1,075; T2: n = 742

**Age Validation (CLDS)**  
Young Adults (19-30)  
T1: n = 3,207; T2: n = 2,052

**Age Validation (CLDS)**  
Middle-aged Adults (31-65)  
T1: n = 15,758; T2: n = 12,697

**Age Validation (CLDS)**  
Older Adults (≥66)  
T1: n = 948; T2: n = 955

Primary Data Processing

**Training Set (70%)**  
n = 125,914  
Model Development

**Testing Set (30%)**  
n = 53,963  
Internal Validation

Feature Selection & Model Development

**Regression Model**: Recursive Feature Elimination (RFE) with Multiple Linear Regression  
**Classification Model**: Logistic Regression, Random Forest, Support Vector Machine  
**Evaluation**: 10-fold Cross-validation, ROC Analysis, Brier Score, Decision Curve Analysis

Resulting Stratified Screening System

**Tier 1: Rapid Screening (4 items)**  
C18 "I feel sad" + C09 "I think my life has been a failure" +   
C06 "I feel depressed" + C19 "I feel that people dislike me"  
*AUC = 0.982, Sensitivity = 0.945, Specificity = 0.926*

**Tier 2: Enhanced Assessment (9 items total)**  
Above 4 items + C12 "I feel unhappy" + C07 "I feel that everything I do is an effort" +   
C14 "I feel lonely" + C02 "I don't feel like eating" + C01 "Things bother me"  
*R² = 0.957 (predicts full CES-D-20 score)*

External Validation Results

**Cross-sample Validation**  
Consistent performance across all validation samples:  
R² > 0.94, AUC > 0.97, Accuracy > 0.91  
Optimal Clinical Utility in Risk Threshold Range (0.3-0.6)
